# Focus on African diversity confirms complexity of skin pigmentation genetics

**DOI:** 10.1186/s13059-018-1395-3

**Published:** 2018-01-31

**Authors:** Tina Lasisi, Mark D. Shriver

**Affiliations:** Department of Anthropology, Pennsylvania State University, University Park, PA 16802 USA

## Abstract

Renewed focus on African populations confirms the complexity of skin pigmentation genetics, and suggests future directions for pigmentation research.

## Introduction

The recent paper by Martin et al. [1] on the genetic architecture of skin pigmentation in KhoeSan populations provides unique insights into the complexity of this phenotype with implications for the broader evolutionary history of skin color around the world. These indigenous South African populations are of particular interest due to their early (approximately 100,000 years ago) divergence from other populations and their light pigmentation relative to equatorial Africans. Along with another recent paper by Crawford and colleagues [2] on skin color diversity in populations from Tanzania, Ethiopia, and Botswana, the work of Martin et al. begins to rectify a bias in the populations traditionally used for pigmentation genetic studies.

Populations in Africa have more genetic diversity than non-African populations, yet this diversity remains underrepresented in studies of the evolutionary genetics of quantitative traits. For example, although skin pigmentation has long been known to be most variable in African populations [3], our current understanding of the genetic architecture of skin pigmentation is based almost exclusively on the study of Eurasians and African/European admixed populations. Moreover, a focus on derived alleles in populations strongly affected by selection for lighter skin in low ultraviolet radiation (UVR) regions may have contributed to the mistaken idea that skin pigmentation is predominantly determined by a small number of single nucleotide polymorphisms with large effects [2].

## Complex and polygenic traits: replicated loci, new loci, and unexplained variance

Using genome-wide association testing and targeted resequencing, Martin and colleagues replicate allelic associations initially discovered in African/European admixed populations and reveal new loci with alleles associated with constitutive skin pigmentation as measured by reflectance spectrophotometry. The replicated loci include: *SLC24A5*, *SLC45A2*, and *KITLG*. However, most other previously identified loci do not replicate significantly in this sample. Non-replications such as these are either the result of low functional-allele frequency and small sample sizes, or epistatic (more pigmented than expected) or hypostatic (less pigmented than expected) interactions with functional alleles at other loci. If the frequency of interacting alleles is different in this population compared with the populations in which the alleles were discovered, there might not be enough statistical power to show the effects of the allele in question, even if this allele is not rare. A novel association at *SNX13* was also identified where a common derived allele in this gene is associated with lighter skin pigmentation. Despite the replication of known pigmentation loci and the association of new genetic variants with skin color variation, a large fraction of the phenotypic variance in these KhoeSan populations remains unexplained, possibly due to the polygenic nature of this highly heritable trait.

As the sample in the study comprises KhoeSan individuals with varying degrees of admixed European and West African ancestry, Martin et al. additionally analyzed the relationship between ancestry and skin pigmentation. As expected, European and West African ancestry proportions are correlated with lighter and darker skin, respectively. Additionally, they also found that KhoeSan persons with high levels of West African ancestry are more variable in their skin pigmentation than KhoeSan persons with high levels of European ancestry. This pattern of higher variability with West African ancestry is likely tied to the presence of higher variance in skin pigmentation in equatorial populations relative to populations living at higher latitudes.

## High variance in equatorial populations: clues on the evolutionary history of skin color?

The distribution of average skin reflectance has guided much of our thinking on how natural selection may have acted to shape skin color variation across populations. However, patterns in the distribution of the variance in skin pigmentation reveal equally telling clues. Martin et al. performed analyses of previously published melanin index (M index: a simple reflectance-based measure of skin pigmentation) data on a global sample of populations which show that lighter-skinned populations at higher absolute latitudes have a narrower distribution of skin pigmentation than darker-skinned populations living closer to the equator. For example, Ghanaians have a variance that is ten times higher than Irish. These differences in variance may be indicating the action of different UVR-related selective pressures on populations.

Although persons with lighter skin pigmentation can efficiently produce vitamin D, they are less able to protect their skin from sunburn and skin cancer or their folate from photolysis. Thus, there are two opposing selective forces acting on skin pigmentation. However, the narrative of selection for lighter pigmentation in low UVR regions and darker pigmentation in high UVR regions appears to give an incomplete picture of the evolutionary history of this phenotype.

The distribution of variance in M index appears to be consistent with a “melanin threshold” model [4, 5] where selection for dark skin acts decisively until a photoprotective minimum is reached, above which populations are free to maintain a wide variance of pigmentation. However, the low UVR regimes of certain regions may represent a strong selective pressure for reducing pigmentation as much as possible to maximize vitamin D production. Furthermore, population bottlenecks and the intensity of selective sweeps at pigmentation loci in Eurasian populations may explain the decreased genetic diversity compared with African populations.

## Beyond baseline: tackling the complexity of pigmentation as a phenotype

Over the past few decades, much of the work on pigmentation genetics has focused on so-called “baseline” constitutive skin pigmentation, which is usually measured as M index readings on the inner upper arm and is thought to represent an individual’s inherited pigmentation potential in a manner that is minimally influenced by environmental UVR. However, pigmentation is far more complex than the use of one single value implies. The effects of tanning and burning responses, sex, and age, for example, require much more study in order to be modeled adequately in genetic studies of pigmentation. Briefly, other body sites and more informative measures of skin reflectance can and should be used to help describe a person’s skin pigmentation.

Martin et al. show that, in their sample, both sex and age have significant effects on tanning status (reflectance on the wrist minus reflectance on the inner arm), although tanning status shows low heritability in their sample and no association with admixture proportions. This may be related to highly variable UVR exposures overwhelming the genetic factors for this measure of tanning status. There exists a significant body of literature in dermatology and anthropology describing differences in UVR-induced tanning and burning responses within and between populations (e.g., Wagner et al. [6] where UVR dose–response curves were calculated). However, there is a distinct lack of data on the heritability and genetic architecture of tanning and burning responses, which is likely due to the extra effort required to measure skin responses functionally and accurately.

Additionally, further work is needed to establish accurate and reliable constitutive skin color measures. For their global comparison of skin pigmentation and latitude, Martin et al. extracted skin reflectance measures from the literature that were made with two reflectometers, which employ different approaches to measuring skin color. Although they are manufactured by one company (Cortex Technology, Hadsund, Denmark) and both refer to their readings as “M index”, one uses a red light-emitting diode (LED) to produce a narrow peak centered on 655 nm (DermaSpec) and the other uses white LED illumination extracting the R component of the tristimulus red, green, blue (RGB) color system as a measure of reflected red light (DSMII). Though these measures are similar, there are clear differences that should be accounted for in analyses that combine data from more than one device.

Furthermore, pigmentation genes likely influence more than just skin. For example, the derived rs1800404 allele in *OCA2*, which has a frequency of 0.65 in KhoeSan, does not appear to be significantly associated with skin pigmentation. However, the effect of this allele may rather be expressed in the range of brown to green eyes found in KhoeSan individuals. Combining quantitative methods for studying skin pigmentation with quantitative methods for studying iris and hair color may thus provide a more complete picture of pigmentation genetics [5].

## Representation matters: understudied populations

Both Martin et al. and Crawford et al. recognize that African populations are underrepresented in skin pigmentation genetics. The majority of such studies have been carried out on individuals of Northern/Western European and East Asian ancestry. This bias in populations studied has consequences on our ability to understand the genetic basis of pigmentation phenotypes as well as their evolutionary history. For example, Eastern and Southern European populations may have experienced different selective pressures relating to constitutive skin pigmentation and skin response related to seasonal variation in UVR. Similarly, little is known about pigmentation in Middle Eastern and South Asian populations, which fall outside simplistic divisions of global populations into European, Asian, and African categories.

The inclusion of underrepresented populations is crucial for a more complete understanding of the evolutionary history of human variation, which includes complex population dynamics. Particular focus on more African populations as well as Austronesian and Melanesian populations (due to their high genetic diversity and early population divergences) will be important for elucidating genetic variability, some of which may have been shared with archaic hominins, and help us understand the evolution of pigmentation and other phenotypes across time-depths. Southeast Asians, indigenous Americans, and Pacific Islanders are other groups who are notably underrepresented in the existing quantitative genetic literature.

These recent studies on the genetic architecture of skin pigmentation in diverse African populations have both confirmed the complexity of African pigmentation genetics and contributed to a more nuanced understanding of skin pigmentation evolution. It is clear that the next steps will have to focus on utilizing more sophisticated phenotyping methods, larger sample sizes, and additional diverse population samples from Africa and other understudied areas of the world.

## Abbreviations

M index: Melanin index
UVR: Ultraviolet radiation
